# Attendance and Utilization of Antenatal Care (ANC) Services: Multi-Center Study in Upcountry Areas of Uganda

**DOI:** 10.4236/ojpm.2015.53016

**Published:** 2015-03-25

**Authors:** Peter Chris Kawungezi, Douglas AkiiBua, Carol Aleni, Michael Chitayi, Anxious Niwaha, Andrew Kazibwe, Elizabeth Sunya, Eliud W. Mumbere, Carol Mutesi, Cathy Tukei, Arabat Kasangaki, Sarah Nakubulwa

**Affiliations:** 1 School of Medicine, Makerere University College of Health Sciences, Kampala, Uganda; 2 School of Health Sciences, Makerere University College of Health Sciences, Kampala, Uganda; 3 School of Biomedical Sciences, Makerere University College of Health Sciences, Kampala, Uganda; 4 Department of Dentistry, Makerere University College of Health Sciences, Kampala, Uganda; 5 Department of Obs & Gyn, Makerere University College of Health Sciences, Kampala, Uganda

**Keywords:** Antenatal Care, ANC, Utilization, Attendance

## Abstract

**Introduction:**

Globally every year 529,000 maternal deaths occur, 99% of this in developing countries. Uganda has high maternal and neonatal morbidity and mortality ratios, typical of many countries in sub-Saharan Africa. Recent findings reveal maternal mortality ratio of 435:100,000 live births and neonatal mortality rate of 29 deaths per 1000 live births in Uganda; these still remain a challenge. Women in rural areas of Uganda are two times less likely to attend ANC than the urban women. Most women in Uganda have registered late ANC attendance, averagely at 5.5 months of pregnancy and do not complete the required four visits. The inadequate utilization of ANC is greatly contributing to persisting high rates of maternal and neonatal mortality in Uganda. This study was set to identify the factors associated with late booking and inadequate utilization of Antenatal Care services in upcountry areas of Uganda.

**Method:**

Cross-sectional study design with mixed methods of interviewer administered questionnaires, focus group discussions and key informant interviews. Data was entered using Epidata and analyzed using Stata into frequency tables using actual tallies and percentages. Ethical approval was sought from SOM-REC MakCHS under approval number “#REC REF 2012-117” before conducting the study.

**Results:**

A total of four hundred one were enrolled with the majority being in the age group 20 – 24 years (mean age, 25.87 ± 6.26). Health workers played a great role (72.04%), followed by the media (15.46%) and friends (12.50%) in creating awareness about ANC. A significant number of respondents went to TBAs with reasons such as “near and accessible”, “my husband decided”, and “they are the only people I know”. 37.63% of the respondents considered getting an antenatal Card as an importance of ANC. 71 (19.67%) respondents gave a wrong opinion (late) on booking time with reasons like demands at work, no problems during pregnancy, advised by friends, just to get a card, long distance and others didn’t know. Almost half of the respondents never knew the recommended number of visits. Religion, occupation, level of education, and parity were found to influence place of ANC attendance, number of ANC visits and booking time. Husbands were necessary to provide financial support, accompany their wives ANC clinic, and ensure that they complete the visits. But their response was poor due to: fear of routine investigations and constrained economically.

**Conclusion:**

The study findings show the actual rural setting of ANC services attendance and utilization. Much sensitization has to be done specifically in these rural areas to empower pregnant women and their husbands as to improve ANC attendance and utilization.

## 1. Background

### 1.1. Maternal Mortality

Globally every year 529,000 maternal deaths occur, 99% of this in developing countries [1]. Uganda has high maternal and neonatal morbidity and mortality ratios, typical of many countries in sub-Saharan Africa. Recent findings from Uganda reveal a maternal mortality ratio of 435:100,000 live births and neonatal mortality rate of 29 deaths per 1000 live births; these still remain a challenge [2].

### 1.2. ANC Attendance and Utilization

Countries with good indicators in maternal and infant mortality have pregnancy related complications identified and managed early, however according to UBOS the overall one time antenatal attendance in Uganda was found at 94% with women in rural areas being twice less likely to attend ANC than the urban women.

According to the report only 8% of rural women in Uganda received ANC from a doctor. Regionally Southwestern Women were more likely to receive skilled care (20%), than Eastern women (3%), while only 2% of the women in Karamoja were reported to seek the same. It was reported that women in Uganda tend to seek antenatal care very late—37% attending for the first time at 6 months or more [2].

Globally, developing countries still face a challenge of poorly implemented ANC programs with irregular clinical visits and long waiting times plus poor feedback to the women [3]. A study in Hadiya zone, Ethiopia found that majority of the mothers who attended ANC did not receive adequate number of visits and initiated the visits later than recommended by the World Health Organization [4].

A similar study done in Nigerian teaching hospital found that Nigerian women tended to obtain care late in pregnancy, and about one third the care was inadequate with almost half (47 percent) of women attending the ANC clinic in the third trimester [5].

The ANC Service utilization in Ethiopia was significantly influenced by maternal age, where mothers aged between 25 – 29 years were less likely to utilize ANC service than women who were 35 years and older. Positive husband attitude towards ANC was also significantly related to ANC service utilization [4] [6].

Mothers’ level of education influenced the use of ANC for which Mothers with primary educational level were more likely to attend ANC than women who are unable to read and write. This study further revealed that availability of women’s time is important as women spend more time on their multiple responsibilities for care of children, collecting water or fuel, cooking, cleaning, and trade than on their own health [7]. In Hadiya; Ethiopia, Family size was a strong determinant of ANC service utilization with greater household size limiting the use of ANC service [4].

A study done by Simkhada B., *et al.* also included maternal education, husband’s education, marital status, availability, cost, household income, women’s employment, media exposure and having a history of obstetric complications. But not leaving out Cultural beliefs, Parity and ideas about pregnancy. Whilst women of higher parity tend to use antenatal care less [8].

Another study done in Ibadan, Nigeria revealed that Women who were Muslims or other religions were more than 2 times likely to attend ANC clinic than women who were Christians. The same study showed that Women who were 25 years and older utilized ANC more than women who were below 25 years of age which agrees with study made in Bangladesh [9] [10].

A study done in rural Local Government Area in Ogun State, Nigeria, identified that women preferred TBAs for various reasons which included: cheap easily accessible culturally acceptable services and more compassionate care than orthodox health workers, and for some it was the only maternity they knew. However some respondents acknowledged that complications could arise from TBA care [11]–[13].

In many countries, TBAs are an important source of social and cultural support to women during childbirth and due to economic constraints, and the difficulty in posting trained professionals to rural areas; many women continue to deliver with TBAs [14]–[17].

A study done rural Uganda revealed ANC attendance being irregular with few women appreciating the fact that ANC attendance was to monitor both the growth of the baby and the health status of the woman. This study also identified Parity as significantly influencing ANC attendance, but level of education, religion and marital status did not.

Several factors influenced Ugandan women ANC seeking behavior which included: perceived high cost of (ANC services, conducting a delivery and treatment), and perceived inadequacy of services provided by the formal health system [18]. Another study in India economic disparity along with cultural belief and restrictions determined care seeking behavior and utilization of health care, resulting in slow decline of child mortality rate [19]. The recent Uganda Maternal Health review revealed that access to the basic antenatal care services has significantly declined [20].

The ministry of health, Uganda in adherence of WHO recommends a simplified antenatal care of four visits; First visit: occurring in the first trimester, between (10 – 20) week of pregnancy, Second visit: scheduled close to week 26 (20 – 28) of pregnancy, Third visit: occurring in or around week 32 (28 – 36) of pregnancy, and lastly Fourth visit (final visit): taking place between weeks 36 and 38 (>36) of pregnancy [3] [21].

This study was set to identify the factors associated with late booking and inadequate utilization of Antenatal Care services in upcountry areas of Uganda. With specific objectives: to describe the knowledge, attitude and practices of women regarding ANC booking; to identify the socio-demographic, obstetric and cultural factors associated with late booking; to describe the knowledge, attitude and practices of women regarding utilization of ANC; to identify the socio-demographic, obstetric and cultural factors associated inadequate utilization of ANC services.

## 2. Methodology

### Study design

This was a cross sectional study using mixed methods approach. Interviewer administered questionnaires, focus group discussions and key informant interviews were employed to capture the required information.

### Study setting

Participants were randomly selected from pregnant women attending ANC Clinic at the selected MakCHS COBES sites until the required number was attained. Data was collected from. These were: Dokolo Health Centre IV in the north, Rwashamaire Health Centre IV in the south west, Kayunga Hospitalin central, and Budadiri Health Centre IV in the east.

Dokolo Health Centre IV is located in northern region of Uganda, Budadiri Health Centre IV is located in eastern region of Uganda, Kayunga district Hospital is located in central region of Uganda, and Rwashamaire Health Centre IV is located in Ntungamo district in western region of Uganda.

### Inclusion criteria

Pregnant women attending ANC clinic at the study site that consented to the study.

### Exclusion criteria

pregnant women who were too sick to participate in the study.

### Sample size calculation

Sample size, *n* was calculated using Kirsh and Leslie formula:

$$n=\frac{t^{2}\times p(1-p)}{m^{2}}$$

where:

*t* = confidence level at 95% (standard value of 1.96);
*p* = 41% women had late booking for ANC;
*m* = margin of error at 5% (standard value of 0.05).

$$n=\frac{1.96^{2}\times 0.41(1-0.41)}{0.05^{2}}$$

*n* = 371.713216; ~372. However, for convenience we enrolled 100 participants from each site [22]–[24].

### Sampling procedure

A systematic random sampling was done using the ANC clinic attendance register, selecting every third pregnant woman. Qualitative data was obtained through FGDs with randomly selected pregnant women attending the ANC clinic and KIs with two TBAs and two health workers at the ANC clinic.

### Data handling

Quantitative data obtained using a questionnaire was entered onto computer using EpiData 3.1 software by double entry, followed by data cleaning and validation. Data was the exported to stata, statistically analyzed and summarized in frequency tables using actual tallies and percentages. Qualitative data initially was analyzed to identify themes and categories.

### Ethical approval

This study was approved by School of medicine Research and Ethics committee (SOMREC) Makerere University College of Health sciences. This was under approval number “#REC REF 2012-117”.

## 3. Results

Four hundred one respondents were enrolled with one hundred (24.94%) at Dokolo health center IV, one hundred (24.94%) at Budadiri health center IV, one hundred and two (25.44%) at Kayunga district hospital and ninety nine (24.69%) at Rwashamaire health center IV.

### 3.1. Demographic Characteristics

As can be seen from Table 1, majority were within 20 – 24 years, with mean age of 25.87 ± 6.26. Predominantly, the respondents were primary school dropouts.

### 3.2. Awareness of Participants about ANC (Figure 1)

Health workers played a great role in creation of awareness (two hundred nineteen, 72.04% of the respondents). The media and friends also played a role with forty seven (15.46%) and thirty eight (12.50%) of the respondents respectively.

### 3.3. Previous Place of Delivery (Table 2)

Though the majority of the respondents had delivered from health facility, a considerably high proportion delivered from home and TBA with reasons that included: fear of hospital mode of delivery, encouraged by mother, husband’s decision, and no pregnancy problems. However some seemed interested in delivering at the health facility but were hindered by: loss of antenatal card, abrupt onset of labor, lack of transport, bad weather like too much rain, distance, and onset of labor at night.

### 3.4. ANC Importance (Figure 2)

Of the majority that considered ANC important, nine (2.47%) thought it was important only to get antenatal Card.

### 3.5. Opinions No ANC Booking (Figure 3)

Much as majority of the respondents had a correct opinion, a good number gave a wrong opinion with reasons such as: work, have no problems during pregnancy, heard from friends, just to get a card, long distance, and to be checked for malaria. Also a considerable number of respondents did not know.

### 3.6. Recommended Number of ANC Visits (Figure 4)

Although the majority of the respondents thought that the recommended number of ANC visits was four, some respondents gave a wrong opinion and others did not know.

### 3.7. ANC Utilization (Table 3)

Much as majority of the respondents received prophylaxis for malaria, anemia and intestinal worms, some never knew why they did so. 12% (48/392) respondents did not sleep under mosquito nets with reasons such as; mosquito nets being expensive, inconveniencing (suffocates, smells bad and makes them feel hot) while for some the nets were torn. Some of the respondents did not receive Tetanus toxoid vaccination with reasons like: never knew that they had to get it, and poor attitude of health workers “they behave badly”.

### 3.8. Associations

In our study, religion influenced place of ANC attendance (Chi = 12.8890, *P* = 0.045) with most of the religious respondents attending from the health facility. Occupation of the respondents also influenced the Place of attendance of ANC (Chi = 14.0202, *P* = 0.007) with all civil servants attending from a health facility.

The level of education also influenced place of ANC attendance (Chi = 8.0923, *P* = 0.231 not significant) with those at tertiary level attending at the health facility, it also influenced place of delivery (Chi = 4.9277, *P* = 0.553). Parity significantly influenced place of delivery (Chi = 18.9506, *P* = 0.000) with most multiparous women delivering from health facility and almost half of the prime gravidas delivering from TBAs’ homes.

More grand multiparous respondents compared to those who were not, delivered from their homes. Occupation also influenced number of ANC visits (Pearson chi = 14.6903, pr = 0.001) with almost all civil servants completing the four visits and close to half of those self-employed not completing. Parity also influenced number of ANC visits (pr = 0.047) with about half of the prime gravidas not completing the four visits.

Furthermore, booking was found to be influenced by occupation (chi = 8.5066, *P* = 0.014) and level of education (chi = 8.1487, pr = 0.043) with the highest proportion of self-employed and secondary level education booking late.

### 3.9. Qualitative Data

#### 1) Attitude of women towards ANC

The attitude towards ANC is becoming positive due to better outcomes in health of the baby and the mother, though some still consider home deliveries as status quo. Some considered ANC as government policy so they attended in order to fulfill their obligation. For it was considered a waste of time due to protocol and delays at ANC clinics.

#### 2) Role of different stakeholders

The main stakeholders identified here were: Health workers (thought to offer ANC services, Mobilize mothers for ANC, provide mosquito nets, and health education), Husbands (thought to be relevant in: financial support, accompaniment of mothers to ANC clinic, and ensuring adherence to ANC).

However husbands respond poorly due to: fear of disclosure for STIs during routine investigations, constrained economic status, and for some cultures, males think they are not supposed to be involved leaving mostly their old mothers to intervene.

#### 3) Late booking

Late booking was thought to follow: absence of complications, long Distance and terrain, some are restricted by their husbands and shame especially teenagers who are shy and timid.

#### 4) Number of ANC visits

There was no specific response, as they thought anytime they felt a problem they go to ANC clinic and with absence of problems, only once just to get the ANC card.

#### 5) Incomplete ANC visits

Incomplete ANC visits were attributed to: long distance, some are satisfied by first visits (1^st^ and 2^nd^), preoccupied by garden work.

#### 6) Failure to comply and adhere to advice and prophylactic treatment

Poor compliance and adherence to prophylactic medication was thought to be due to: morning sickness, large size tablets like fansidar, and use of native medicines like “mumbwa” a local concoction made of clay and herbs.

## 4. Discussion

As predicted by other studies elsewhere, majority of the respondents’ sensitization (72.04%) was by health workers; if health workers are encouraged and empowered, this would make ANC awareness better. Visiting TBAs for pregnancy care was supported with reasons like: near and accessible, husband’s decision, and the only ones known. These were similar to findings in rural Local Government Area in Ogun State, Nigeria [11].

Delivery at home or TBA was supported with reasons such as; fear of mode of delivery at hospital, encouraged by mother, husband’s decision, and lack of problems during pregnancy. These were similar to findings elsewhere but were more supported by the social and cultural support given by TBAs during child birth [14]–[16]. However some respondents were interested in delivering at health facility but were limited by: loss of antenatal cards, abrupt onset and fast progress of labor, lack of transport, bad weather (too much rain), distance, and wee hours.

Just like in studies elsewhere, ANC was considered to be important in: assessing baby’s position, tetanus vaccination, and treatment when sick [18]. However almost half of the respondents, 37.63% considered getting the antenatal card important as a requirement at time of delivery. This might have contributed to late booking and incomplete visits since what is aimed at is the ANC card. These findings were expected apart from just getting antenatal card.

Work, absence of pregnancy problems, advise from friends, just to get a card, long distance, and malaria checkup; were reasons given for late booking. Most of these were expected, they are similar to the findings in UDHS 2006, and elsewhere [2] [4] [5].

As it was found in Hadiya zone; Ethiopia, majority of the mothers never received adequate number of visits [4]. This might be one of the hindering factors for poor utilization of ANC services and predisposes them to delivery at home or TBAs homes.

In this study, some respondents did not get prophylactic treatment because they never knew why. Others never slept under mosquito nets because of; lack of money, inconveniencing (suffocates, smells bad and they feel hot) and torn nets. Almost half of respondents never received Tetanus vaccination due to: lack of knowledge, and poor health worker attitude one respondent said “they behave badly”. These responses were expected given the setting of our study areas. More sensitization has to done to enlighten mothers why they need prophylactic medication and Health workers need to change their attitude in order to quench their clients’ fears.

Similar to study findings elsewhere; religion, occupation, parity and education level influenced place of ANC attendance, and place of delivery and to some extent these findings were expected [7]–[10]. In this study, religious, civil servants, multiparous and educated mothers attended ANC at health facility and delivered at health facilities. Therefore if women are economically empowered, educated and more emphasis made at different places of worship, this could improve ANC attendance and utilization at health facilities [25]–[27] and positively impact on maternal and child health [27] [28].

Empowering husbands to escort their wives to attend ANC would encourage and motivate many mothers completing ANC visits, adhering to drugs and utilizing other ANC services since this would making planning easier. The good outcome for both babies and mothers has led to positive attire of many mothers towards ANC.

Stake holders identified were: health workers (Mobilize mothers for ANC, providing mosquito nets, and health education) and Husbands (financial support, accompaniment to ANC clinic, and ensuring adherence to ANC services). However husbands responded poorly because of: fear routine investigations, constrained economically, and traditional restriction. To some extent these findings were expected.

Late booking was due to: absence of complications, long Distance and terrain, husband’s restriction and shame for teenagers. Some of the respondents gave a wrong opinion about number of ANC visits due to reasons like: “anytime a problem develops”, “Once, just to get the ANC card”. However Incomplete ANC visits were attributed to: long distance, and work. Some of these findings were expected and have not yet been demonstrated in studies elsewhere.

Poor compliance for prophylactic medication was due to: morning sickness, large size tablets like fansidar, and native medicines like “mumbwa”, which have not yet been studied elsewhere.

## 5. Conclusion

The study findings show the actual rural setting of ANC services attendance and utilization. Much sensitization has to be done specifically in these rural areas to empower pregnant women and their husbands as to improve ANC attendance and utilization. These findings will also provide a basis for improvement and revision of ANC sensitization schemes, ANC provision, and ANC services utilization.

## Figures and Tables

**Figure 1:**
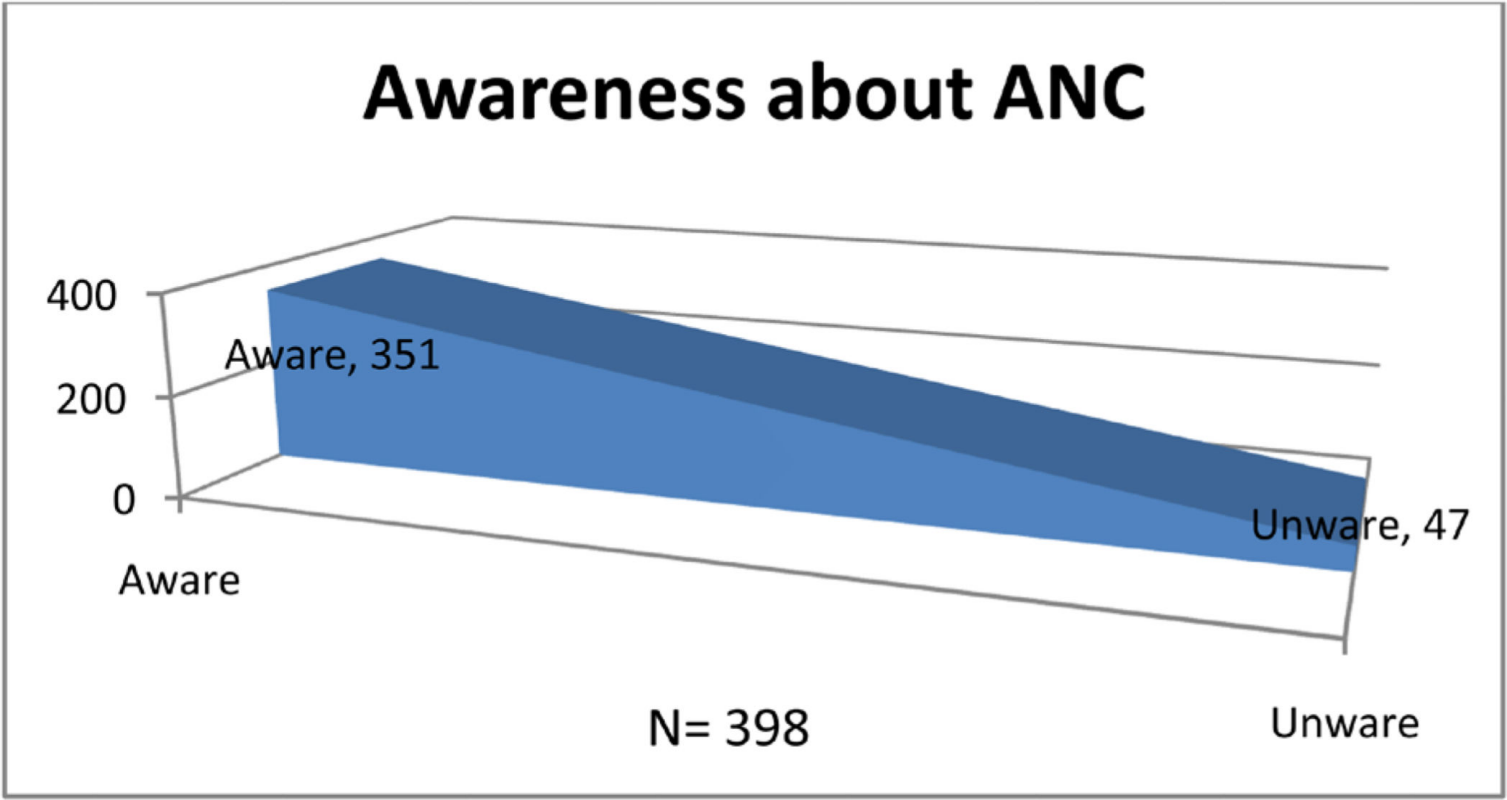
Shows percentage of participants aware of the value of t ANC.

**Figure 2:**
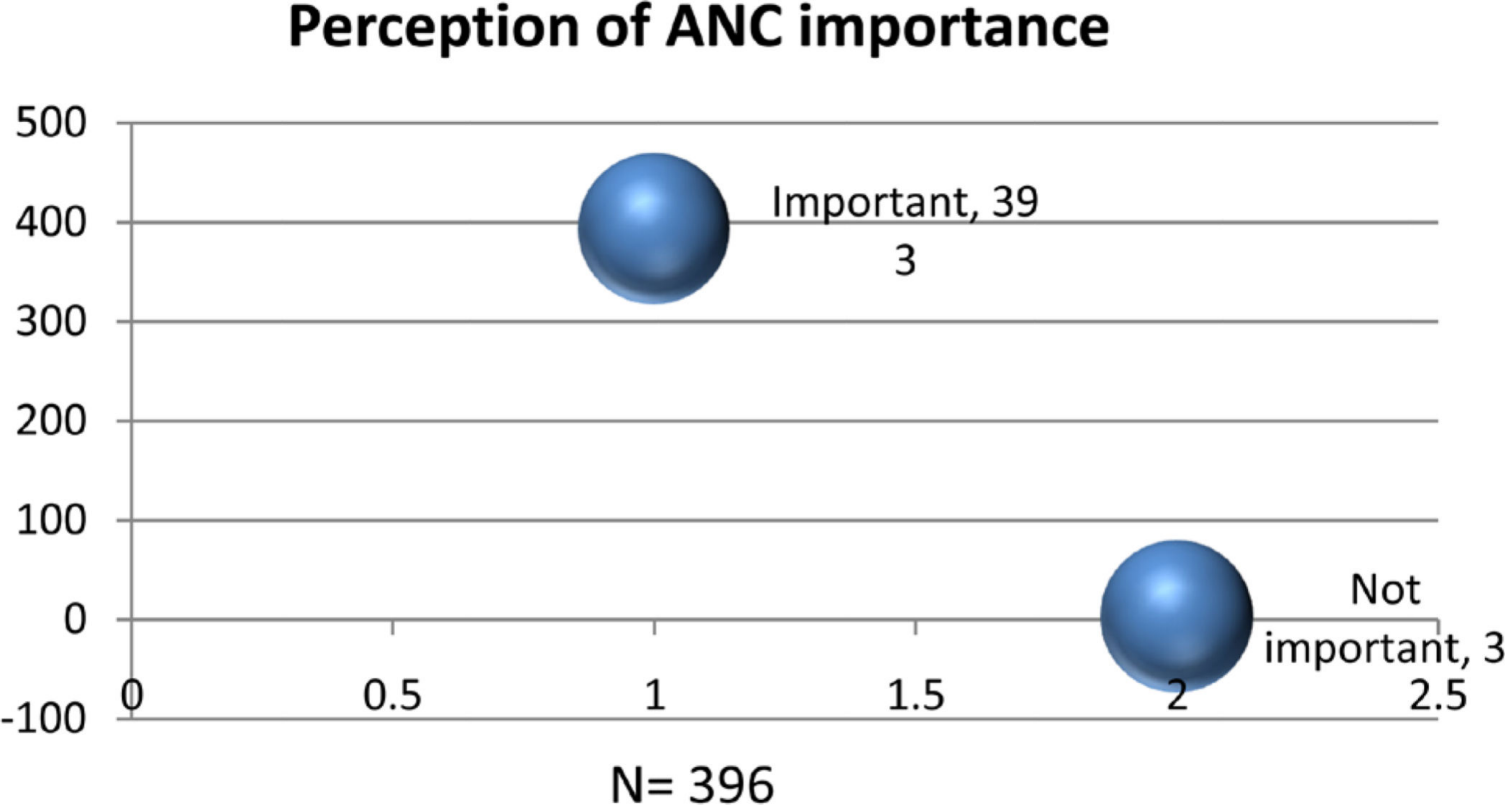
Shows participants perception of ANC importance.

**Figure 3:**
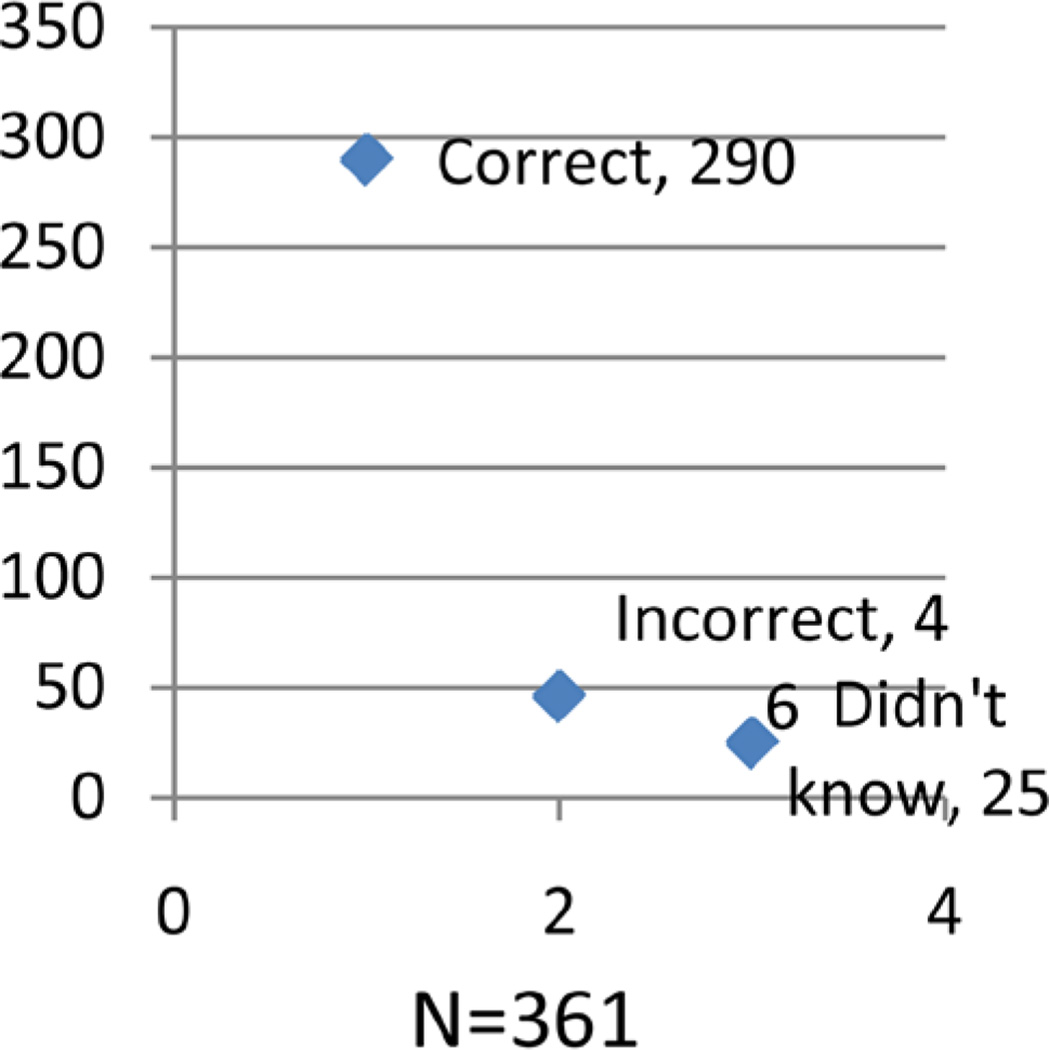
Shows the opinion of participants on ANC booking.

**Figure 4:**
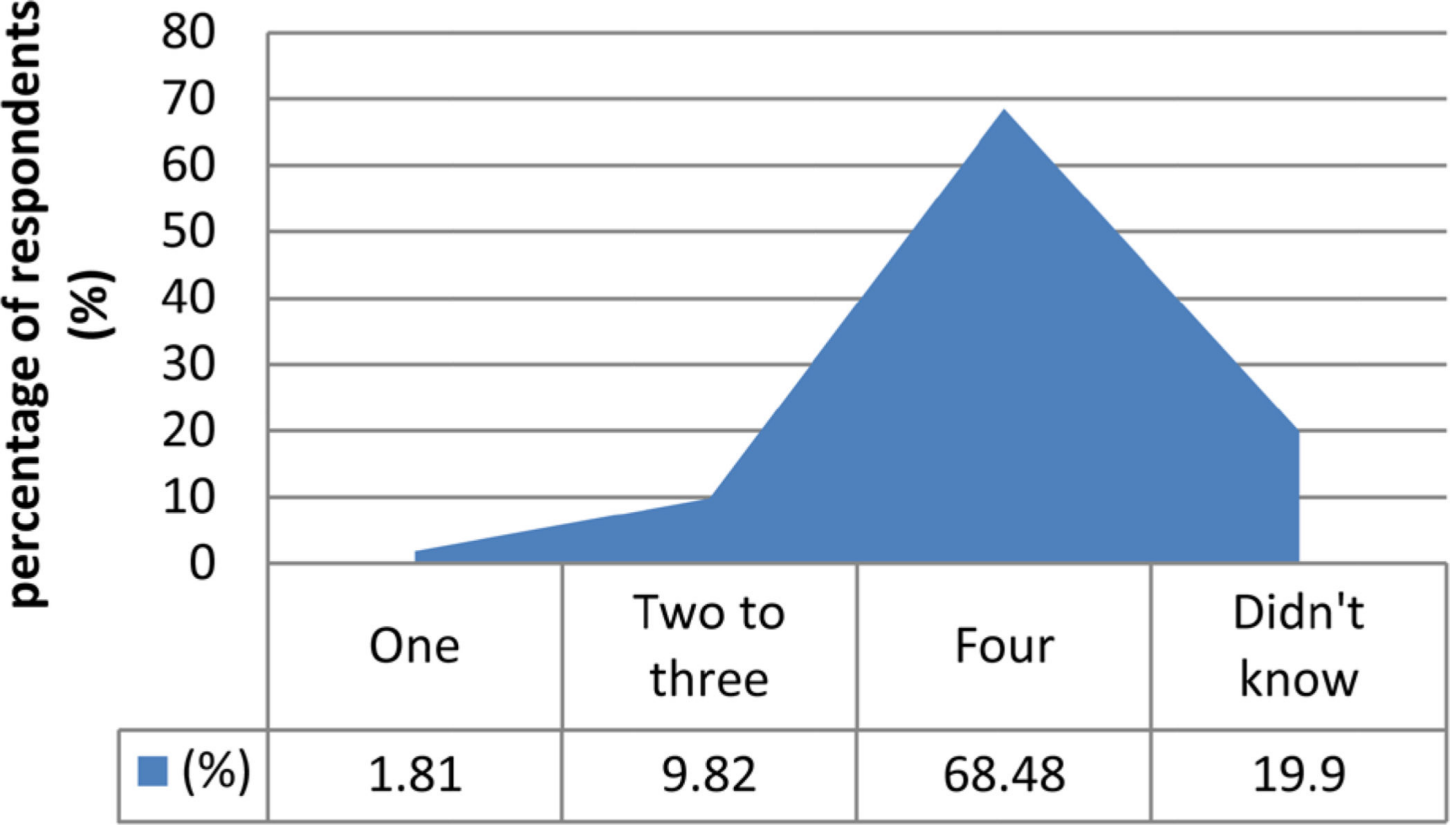
Shows the opinion of participants on recommended number of ANC visits.

**Table 1:** Demographic characteristics of participants in a multi-center study on attendance and utilization of ANC in upcountry areas of Uganda, 2012.

Variable	Frequency	Percentage (%)
**Age**		
<20	53	13.22
20 – 24	146	36.41
25 – 29	86	21.45
30 – 34	63	15.71
>34	53	13.22
Total	**401**	**100**

**Marital status**		
Married	352	88
Single	48	12
Total	**400**	**100**

**Religion**		
Roman catholic	184	49.07
Anglican	117	31.20
SDA	20	5.33
Moslem	54	14.40
Total	**375**	**100**

**Education level**		
None	33	8.38
Primary	229	68.12
Secondary	112	28.43
Tertiary	20	5.08
Total	**394**	**100**

**Parity**		
Prime	59	15.61
Multiparous	319	84.39
Total	**378**	**100**

**Occupation**		
Peasant	268	74.24
Civil servants	20	5.54
Self employed	73	20.22
Total	**361**	**100**

**Table 2:** A table showing place of delivery for the previous pregnancy.

Place of delivery	Frequency	Percentage (%)
Health facility	300	87.21
Home	33	9.59
TBA	11	3.20

**Table 3:** Shows utilization of some ANC components of ANC.

Variable	Frequency	Percentage (%)
**Prophylaxis for malaria (Fansidar)**		
Yes	359	94.97
No	19	5.03
Total	**378**	**100.00**

**Prophylaxis for anemia**		
Yes	325	94.20
No	20	5.80
Total	**345**	**100.00**

**Prophylaxis for intestinal parasites**		
Yes	297	89.46
No	35	10.54
Total	**332**	**100.00**

**Sleep under mosquito net**		
Yes	344	87.76
No	48	12.24
Total	**392**	**100.00**

**Received Tetanus toxoid vaccination**		
Yes	351	90.46
No	37	9.54
Total	**388**	**100.00**

## Acronyms and Operational Definitions

UDHS: Uganda Demographic Health Survey
ANC: Antenatal Care
SOMREC: School of Medicine research and ethics Committee
MakCHS: Makerere University College of Health Sciences
COBES: Community Based Education and Service
TBA: Traditional Birth Attendant
MOH: Ministry of Health
MDGs: Millennium Development Goals
HC: Health Center
FGD: Focus Group Discussion
KIs: Key Informant interviews
Booking: First time ANC attendance during pregnancy
Late booking: First time ANC attendance after first trimester (12Weeks)
Inadequate ANC utilization: Includes less than 4 visits, not taking fansidar, hematinic and Dewormers, not using mosquito nets and not getting tetanus injection among others plus failure to adhere to advise given during ANC visits
Health worker: Cadres including midwives, medical officers, and clinical officers
